# Supplementary material for: The Fe-FeSi phase diagram at Mercury’s core conditions
Source: Nat Commun. 2022 Jan 19;13:387. doi: 10.1038/s41467-022-27991-9 (PMC8770642; doi:10.1038/s41467-022-27991-9)
Supplement: Supplementary file 1 — Supplementary Information [file 41467_2022_27991_MOESM1_ESM.pdf]

Supplemental Discussion 1

A key point of distinction between the present study and previous literature has been the observation of a high temperature fcc-bcc transition, rather than the formation of an fcc + B2 miscibility gap[1,2]. Direct identification of different types of cubic long-range order is critically dependent on the observation of weak superstructure reflections (the DO3 (111) reflection, DO3/B2 (002)/(001) reflection), which can generally be observed upon careful inspection of the image plate. In the present study, B2/DO3 superstructure was observed for 'bcc-like' phases in Fe16Si, Fe22Si and Fe30Si. B2 superstructure reflections were also observed in diffraction patterns of the quenched heating spots of Fe7Si in runs where melting temperatures were achieved. However, these reflections were never observed for Fe7Si at relevant temperatures just below melting. Complementary information can be gleaned from the volume of the here-reported bcc phase alongside literature trends for the hcp, fcc and B2 phases. Shown in Figures S1a and S1b, the volume of fcc Fe7Si is consistent to within 2 GPa with the thermal equation of state of fcc-Fe reported in Tsujino et al.[3] over the pressure range of Mercury's core, indicating that Si alloying does not significantly change the volume of fcc Fe. Deviations occurring at higher pressures are expected due to the progressive spin transition in fcc Fe and the difference in studied pressure range of the present study and Tsujino et al.[3]. Similarly, the elastic and thermal parameters of hcp Fe-Si alloys are largely consistent with those of pure Fe over the pressure range of the present study[4,5,6,7]. The thermal parameters are similar or slightly lower than Fe for the B2/DO3 phases, but ambient temperature volume is influenced significantly by the addition of silicon e.g.[8,9,10]. A synthesis of available literature indicates that for all but the fcc phase of the Fe-Si solid solutions, a Mie-Gruneisen-Debye equation of state with thermal parameters of $\gamma \approx 2$, $q \approx 1$ and $\theta_D \approx 417K$ can provide a first approximation of the thermal pressure of this system, with varying ambient temperature volume and elasticity depending on the structure and composition. In Figure S1a it is shown that the here-assigned bcc phase has a systematically higher volume than the observed fcc phase at similar P-T conditions, as expected for a re-entrant high temperature fcc-bcc transition. Figure S1c shows the volumes of B2 and bcc-structured phases at high temperatures, reduced to 300 K isotherms via the Mie-Gruneisen-Debye thermal parameters stated above. It is shown that the reduced 300 K isotherm of Fe7Si is consistent with an extrapolation of P-V equation of state of the starting composition of the alloy, employing EoS parameters from high pressure DAC and ambient pressure ultrasonics literature after[10]. Despite similarities in isotherms between the present study and Fischer et al.[2] at low reduced pressures (corresponding to experimental pressures of less than 60 GPa), at higher pressures larger discrepancies are observed between the two studies suggesting a large difference in composition between what is reported for this alloy and what has been observed previously. Noteworthy, these observations have been reproduced over three independent experimental runs, with additional diffraction patterns shown in Supplemental Figures S2-S4.

Finally, based on chemical and textural analysis with SEM and FIB, the recovered samples of Fe7Si exhibit melt pools systematically enriched with silicon relative to the surrounding solid in agreement with the compositional bounds observed in literature[11,2]. While some studies have argued for an influence of oxygen on the binary Fe-Si phase diagram, this behaviour is heavily debated[12,13,14]. In the present study, a few samples are observed to exhibit some quantity of SiO_2 in the hotspot, however no FeO or Fe-Si-O signature is observed either by X-ray diffraction or EDX. Subsolidus and melting experiments on alloy Fe5Si show that this SiO_2 signature can be attributed to the reaction of oxidized surface material at the lowest temperatures investigated with laser heating, and the subsequent migration of the resulting SiO_2 at temperatures close to melting as shown in Supplemental Figure S5. As SiO_2 is expected to be relatively inert in equilibrium with Fe-Si alloys, this is not believed to have an effect on the results of the present study. The XRD data collected in the present study have only been done at high temperatures upon temperature increase, as decreasing temperature after achieving melt conditions would chemically re-equilibrate the solid and liquid portions of the sample and prevent the determination of liquid compositions.

Supplemental Table 1

Nominal Composition	Recovered Composition	Recovered Composition Error
Fe-5Si	4.8	*
Fe-7Si	7.1	0.3
Fe-16Si	16	0.5
Fe-22Si	22	0.5
Fe-28Si	28	*
Fe-30Si	30	2

Table 1: Starting compositions of the investigated alloys, with units of wt% Si. Asterisk indicates only one melting run was performed, and so errors due to compositional variation from sample to sample could not be quantified.

0.1 Supplemental Figure 1

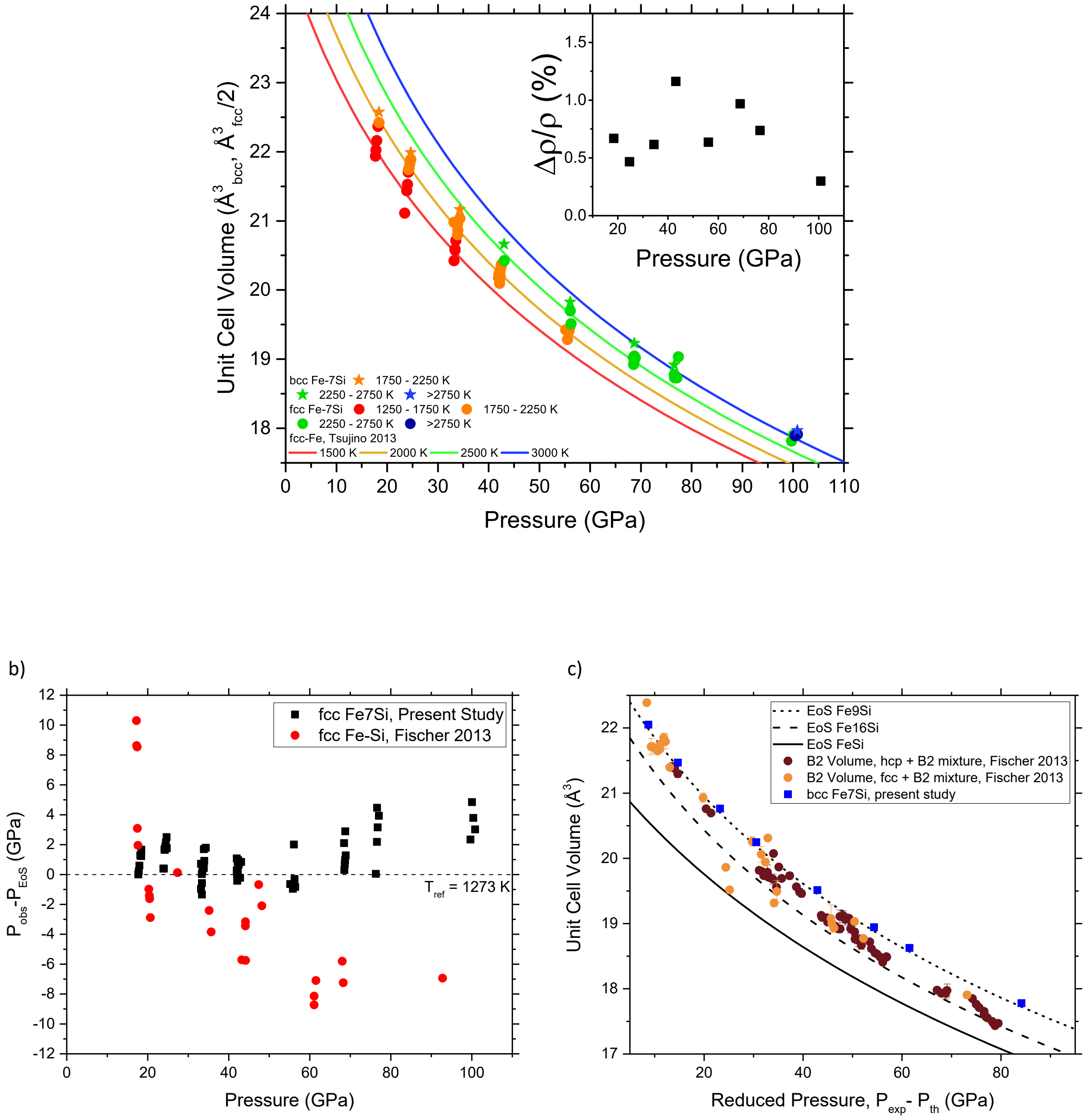

Figure 1: a) Collected volumes of fcc and bcc Fe7Si. The fcc Fe7Si volumes are in excellent agreement with literature equations of state for fcc Fe[3], and the bcc volumes are systematically larger than those of the fcc phase for similar P-T conditions. Inset: Density contrast between fcc and bcc phases at similar P-T conditions. b) Residuals of the difference in pressure from the present study and literature[2], relative to the equation of state of fcc Fe[3]. c) Comparison of reduced 300 K isotherms for the presented bcc phase and previous data on the hcp + B2 and fcc + B2 phases[2]. For details see Supplemental Text S1.

0.2 Supplemental Figure 2

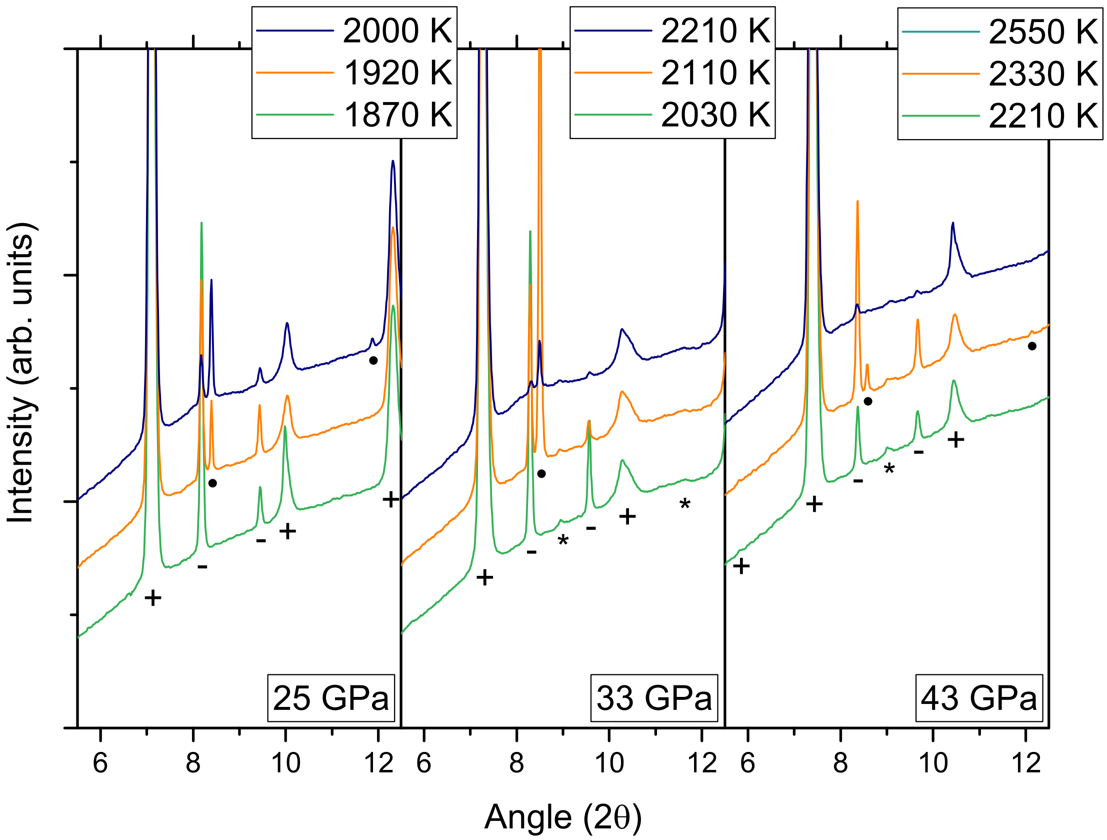

Figure 2: Collected diffraction patterns of Fe-7Si at temperatures close to melting. Green lines indicate patterns before the observation of the bcc phase, orange indicates conditions where the bcc phase was observed, and blue indicates patterns where liquid diffuse scattering was observed. Symbols indicate the phase assignment of the reflections - Crosses: KCl, Bars: fcc Fe-7Si, Closed circles: bcc Fe-7Si and Asterisks: hcp Fe-Si.

0.3 Supplemental Figure 3

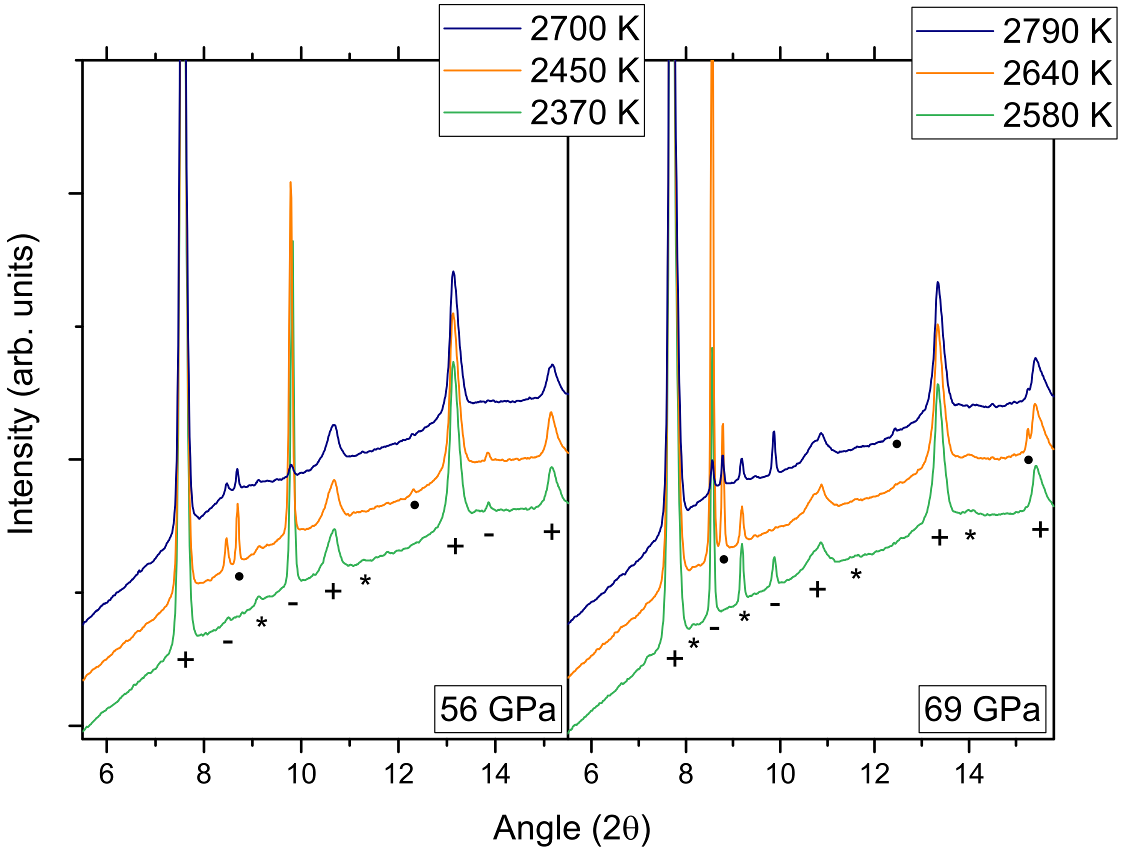

Figure 3: Collected diffraction patterns of Fe-7Si at temperatures close to melting. Green lines indicate patterns before the observation of the bcc phase, orange indicates conditions where the bcc phase was observed, and blue indicates patterns where liquid diffuse scattering was observed. Symbols indicate the phase assignment of the reflections - Crosses: KCl, Bars: fcc Fe-7Si, Closed circles: bcc Fe-7Si and Asterisks: hcp Fe-Si. At these conditions hcp phase volumes are not observed to vary with temperature.

0.4 Supplemental Figure 4

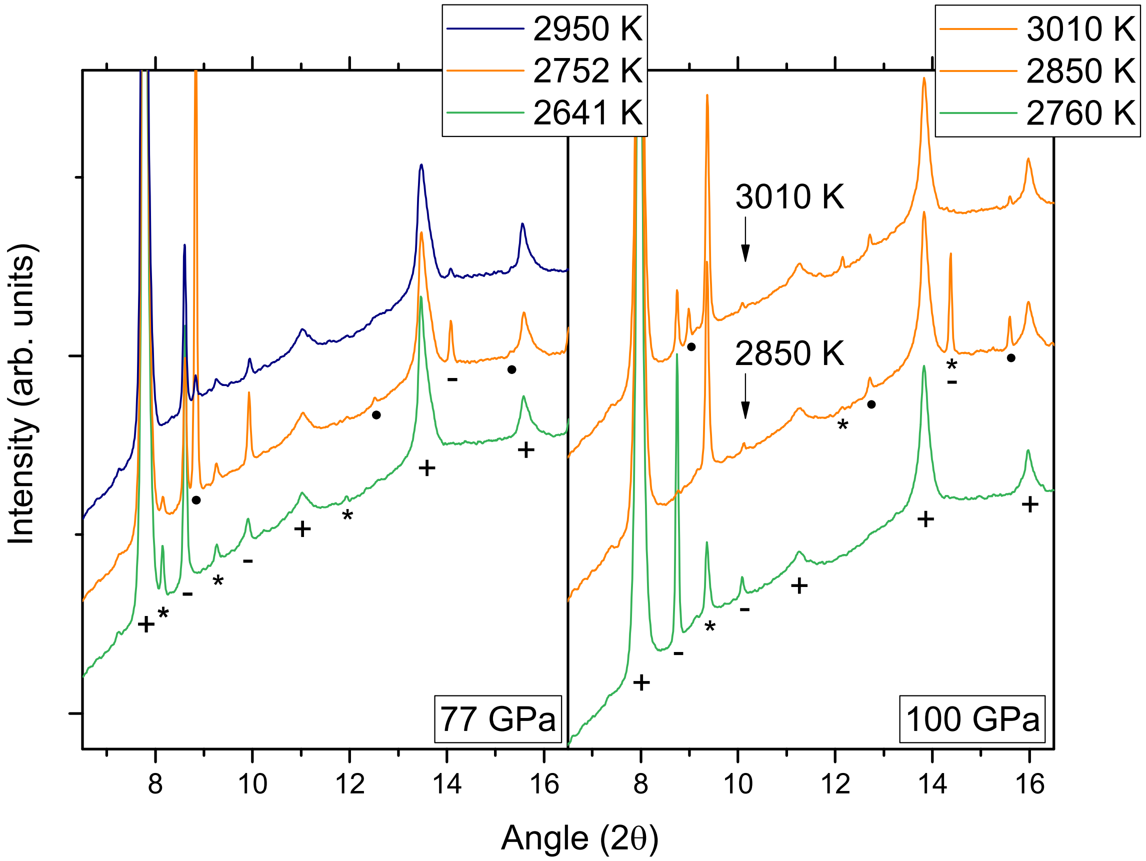

Figure 4: Collected diffraction patterns of Fe-7Si at temperatures close to melting. Green lines indicate patterns before the observation of the bcc phase, orange indicates conditions where the bcc phase was observed, and blue indicates patterns where liquid diffuse scattering was observed. Symbols indicate the phase assignment of the reflections - Crosses: KCl, Bars: fcc Fe-7Si, Closed circles: bcc Fe-7Si and Asterisks: hcp Fe-Si. At these conditions hcp phase volumes are not observed to vary with temperature, however the increasing prominence of these reflections in the heating runs is attributed to the steepening of temperature gradients with thin samples and PTM, alongside the large expansion of the hcp and hcp+B2 stability fields with pressure.

0.5 Supplemental Figure 5

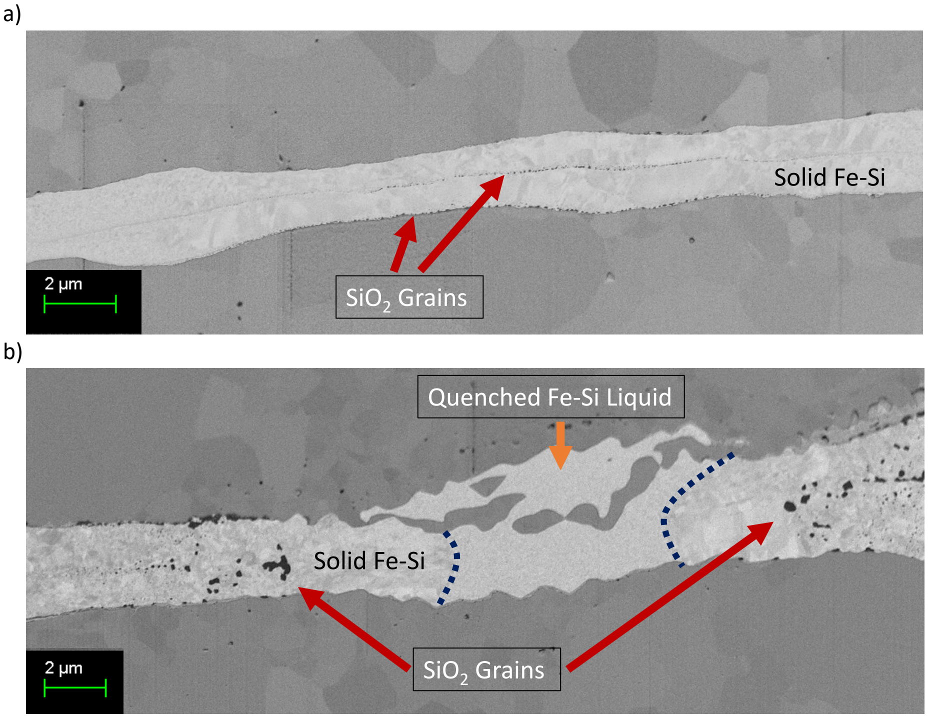

Figure 5: Electron backscatter images of a) Fe7Si quenched at 59+/-5 GPa and 2580 +/- 200 K and b) Fe-5Si quenched at 34+/-3 GPa and 2600+/-200 K. a) At these P-T conditions, melting nor an Fe-Si mixed phase was observed for this sample. It can be seen that small SiO_2 grains line the surface of the alloy, indicating that the SiO_2 signature found in these alloys primarily arises from oxidation of the sample surface during, or after synthesis. b) Fe5Si was observed to melt at these conditions, with solid and liquid compositions which differ by less than 0.2 wt%, below the experimental resolution of EDX. At these P-T conditions small SiO_2 grains formed from surface oxidation coalesce into larger grains and migrate out of the laser heating spot.

0.6 Supplemental Figure 6

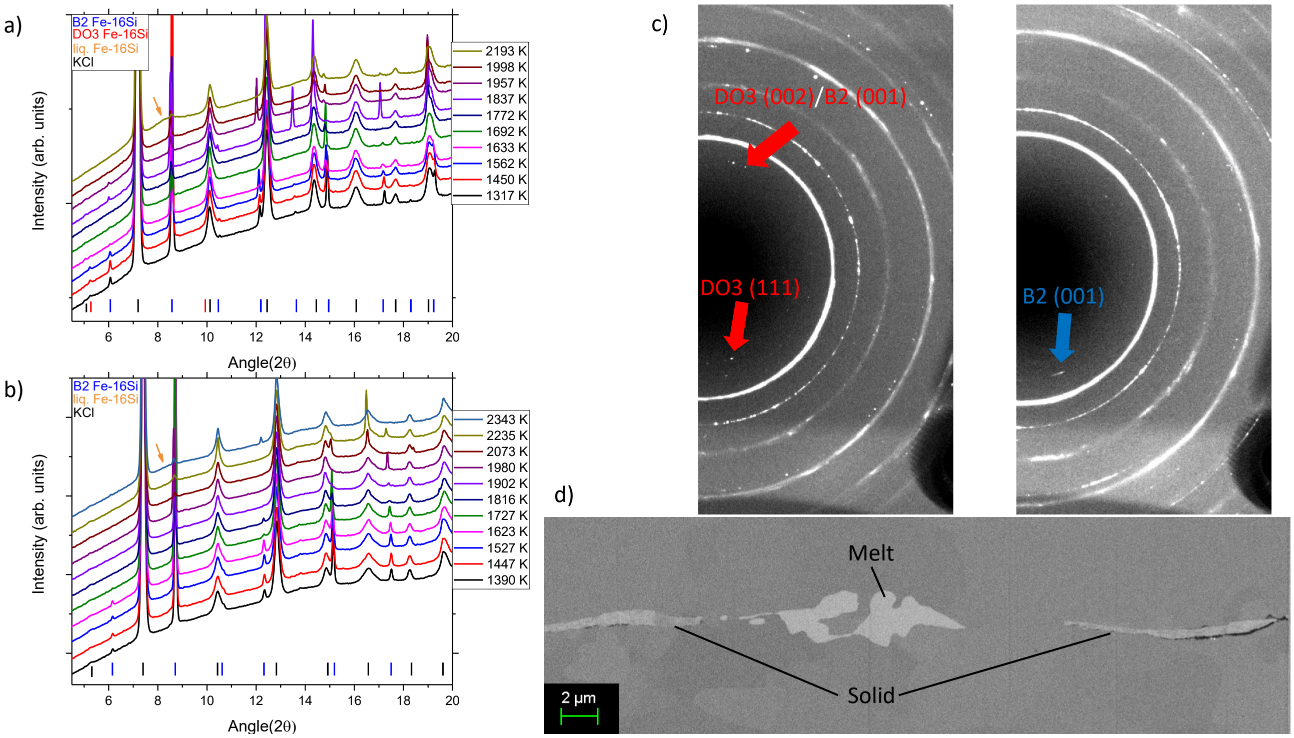

Figure 6: a) Integrated diffraction patterns of Fe16Si at 36(2) GPa and 1320-2190 K. The DO_3 structure is visible at low temperatures. Above 1560 K the DO_3 reflections are lost on both the diffraction pattern and image plate, while B2 reflections remain. b) Integrated diffraction patterns of Fe16Si for increasing temperature at 41(2) GPa and between 1390 and 2350 K. No DO_3 reflections are observed over the temperature range investigated. c) Diffraction images at 27 GPa and 1560 K (left), and 41 GPa and 1570 K showing the DO_3 (111) and the DO_3(002) or B2(001) reflections. d) Electron backscatter image of the quenched Fe16Si alloy after melting at 37(2) GPa showing a texturally homogeneous melt pool and texturally heterogeneous solid.

0.7 Supplemental Figure 7

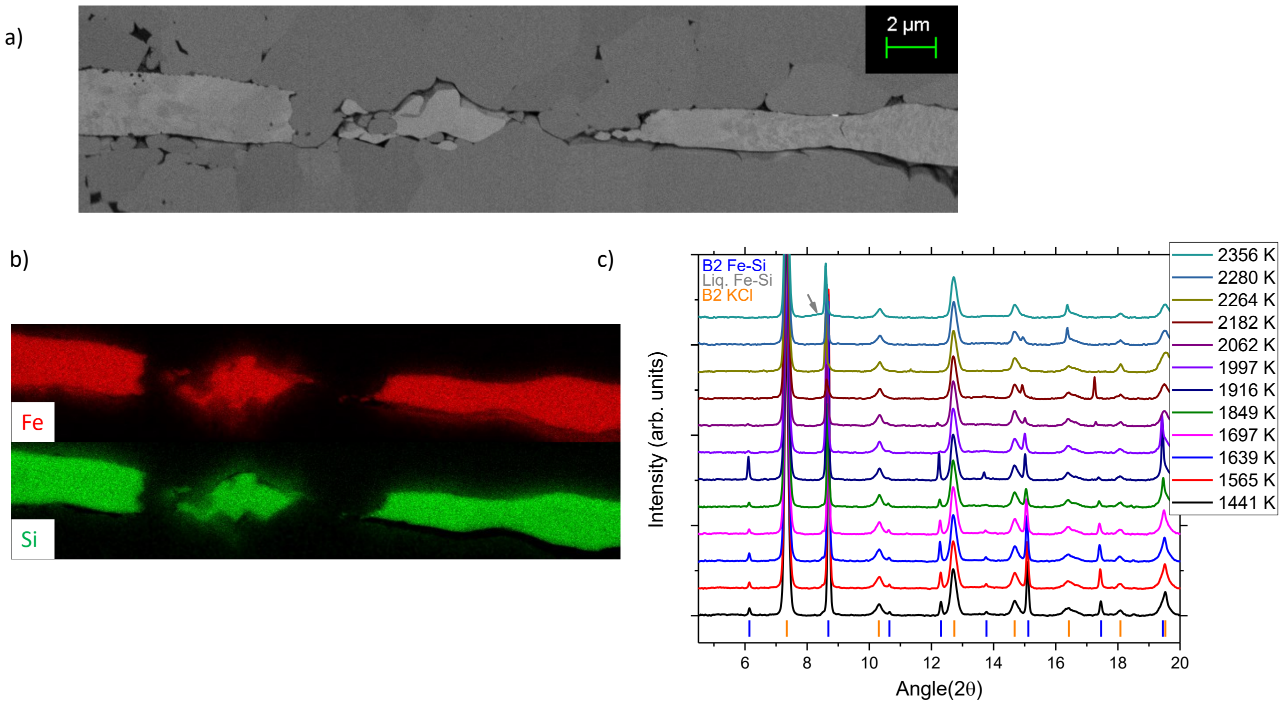

Figure 7: a) Electron backscattered image of a sample of Fe22Si quenched from a molten state at 38 GPa and b) corresponding EDX chemical analysis, in which Si is depleted in the quenched melt (18.5+/-0.5 wt% Si) relative to the surrounding solid (22.5+/-0.5 wt% Si). c) Integrated diffraction patterns of Fe22Si at 38(2) GPa for increasing temperature between 1440 and 2360 K. Only the B2 structure is observed for this composition at the P-T conditions investigated in this study.

0.8 Supplemental Figure 8

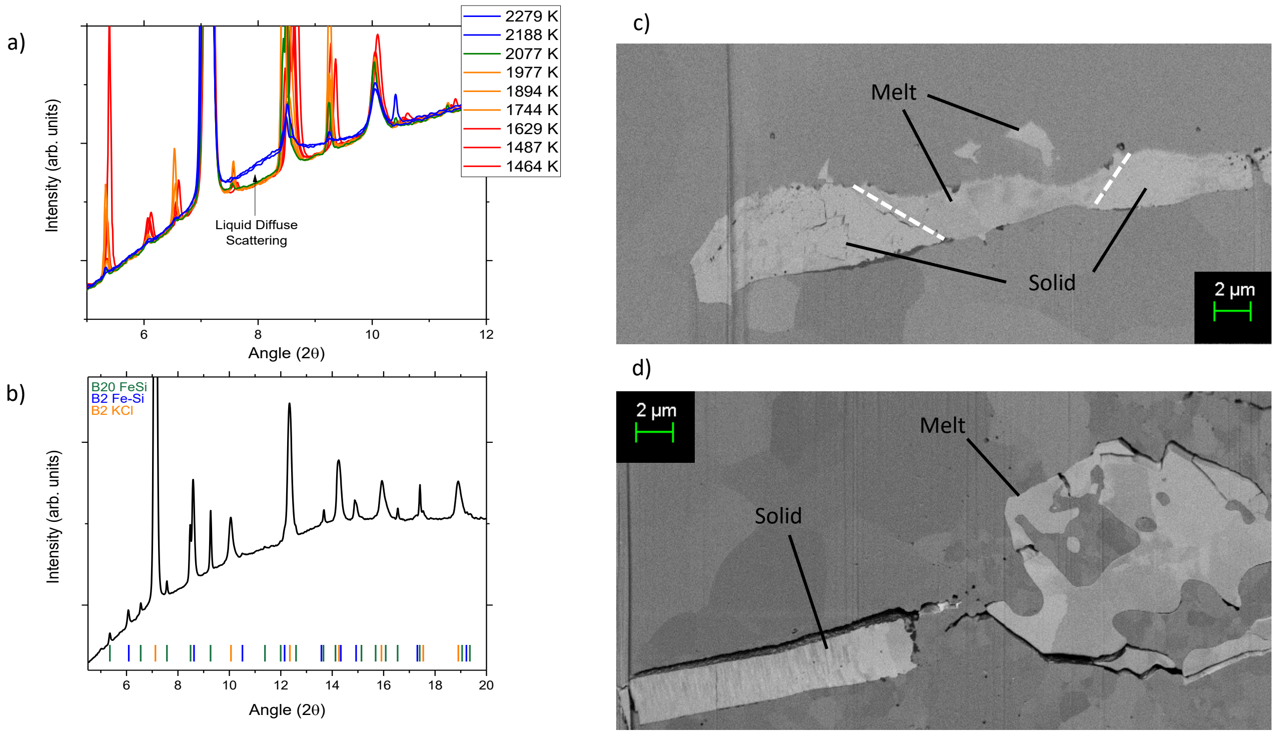

Figure 8: a) Integrated diffraction patterns of Fe30Si showing the appearance of liquid diffuse scattering at high temperatures. A substantial change in background is observed between 2080 K and 2190 K (at 25 GPa) centred near 8.5 degrees, indicative of the onset of melting. b) An integrated diffraction pattern of Fe30Si at 25 GPa and 1630 K displaying the phase assignment of the pattern. All peaks can be attributed to B20 FeSi, a B2 Fe-Si structure or KCl. c) EBS image of a recovered sample of Fe30Si at 23(1) GPa. The quenched melt pool is systematically depleted in Si relative to the surrounding solid (melt: 29.0+/-0.5 wt% Si, solid: 33.5+/-0.5 wt% Si). d) EBS image of an off-line heating experiment performed on Fe28Si at 32(2) GPa. The melt pool is enriched in Si relative to the surrounding solid (melt: 29.1+/-0.6 wt% Si, solid: 28.1+/-0.5 wt% Si).

0.9 Supplemental Figure 9

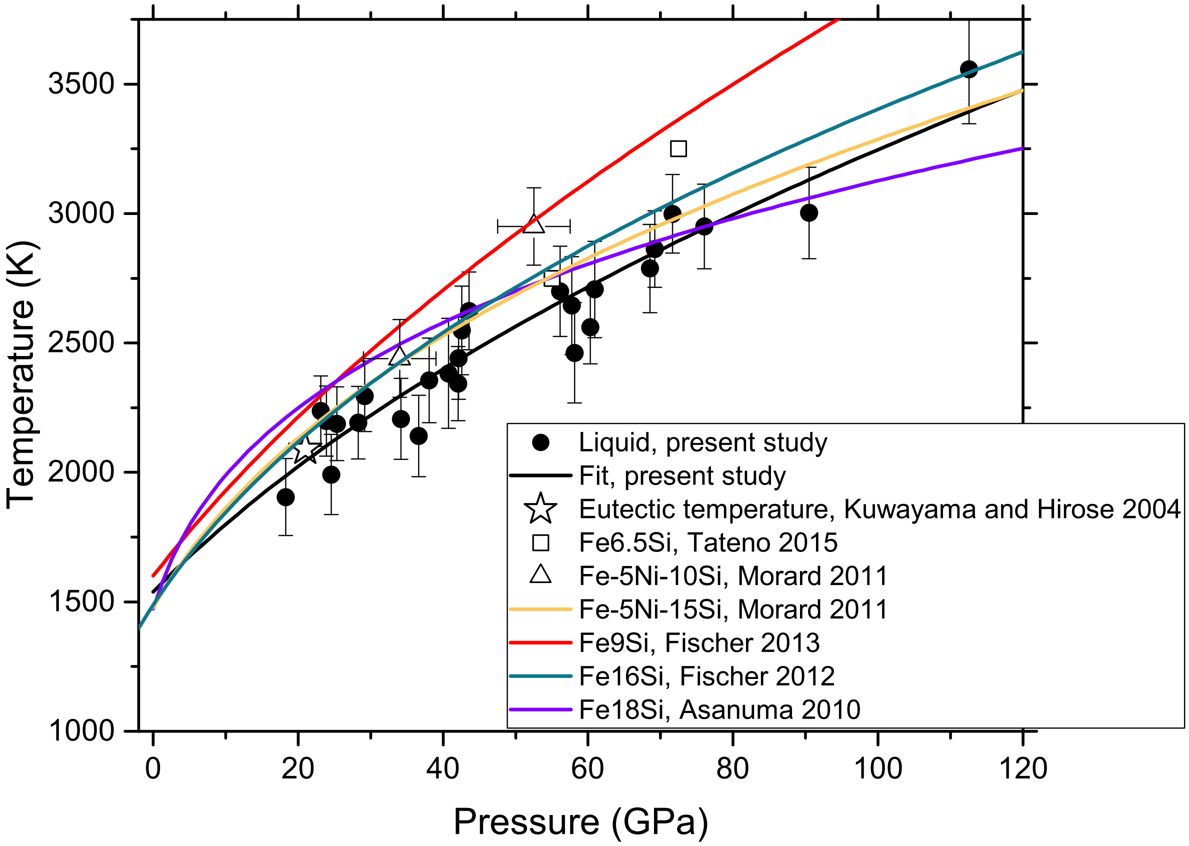

Figure 9: Melting temperature vs. pressure for Fe-Si alloys based on the present study and available literature[2,8,15,16,17]. Symbols represent individual datapoints for compositions and/or studies where a Simon-Glatzel fit was not available.

0.10 Supplemental Figure 10

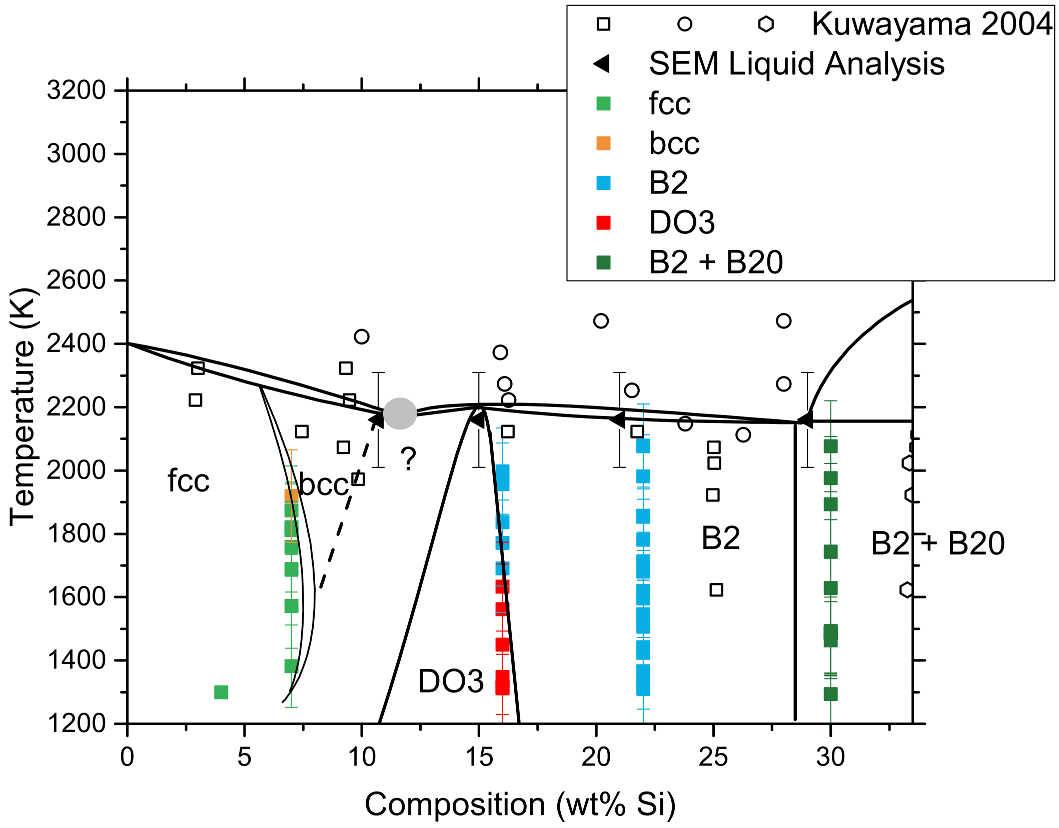

Figure 10: Temperature-composition phase diagram at 25 GPa. Open squares and circles indicate the liquid and solid compositions respectively, as reported in Kuwayama and Hirose [18] at 21 GPa. The presented phase diagram is compatible with the latter datapoints to within experimental uncertainties.

0.11 Supplemental Figure 11

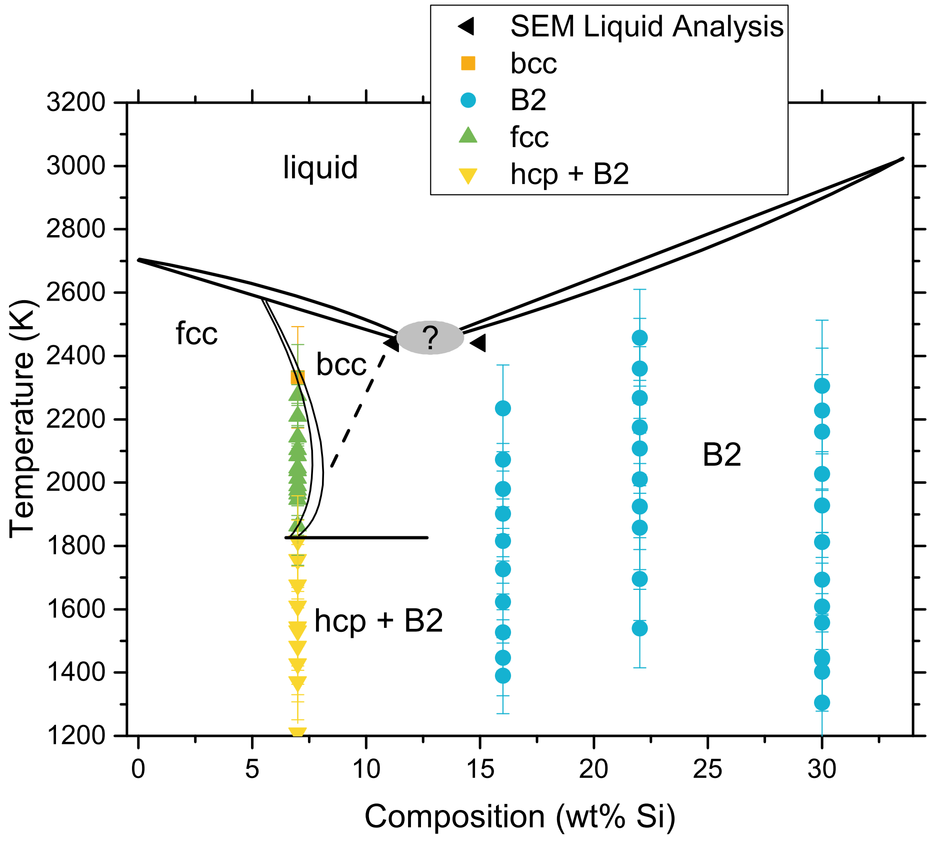

Figure 11: Constructed Temperature-Composition Phase Diagram at 40 GPa, showing the datapoints used to construct the phase diagram in the present study, composed of datapoints within 3 GPa of 40 GPa.

0.12 Supplemental Figure 12

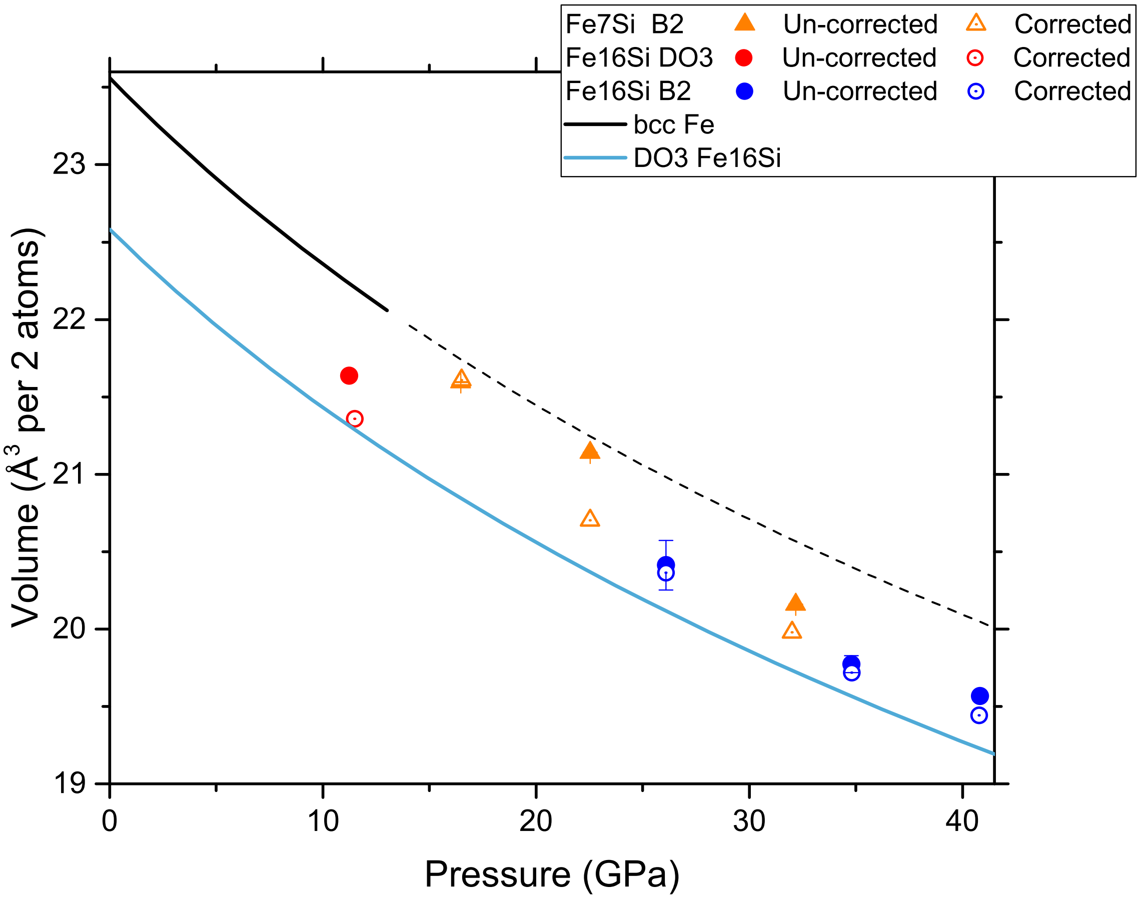

Figure 12: Volume of B2/DO_3 phases observed in the quenched melt from the Fe7Si and Fe16Si alloys, alongside literature equations of state for bcc Fe and DO3 Fe16Si [19,9,10]. With the exception of one point for Fe16Si at 11 GPa, all quenched samples exhibited the B2 structure when quenched from high temperatures over the here-considered pressure range. Fe7Si melted at pressures below about 35 GPa were observed to form a single phase B2 alloy. Between 39-67 GPa, this alloy formed either an hcp + B2 mixture or a metastable hcp+fcc+B2 mixture. Above 75 GPa, the quenched melt of Fe7Si was observed to form a single-phase hcp alloy. While the effects of nonhydrostatic stress are minor at high temperatures due to the relaxation of stress in the sample and KCl pressure medium, the mismatch in thermal expansion of the KCl and the sample, alongside the specific details of the P-T path taken can lead to residual non-hydrostatic stress when the sample is quenched to ambient temperature such that the reported volume is an upper bound for the 'hydrostatic' volume of the alloy [20,21]. This effect appears to be stronger at low pressures. However regardless of whether this effect is taken into account or not, all volumes remain within the Fe-rich side of the Fe-Si phase diagram over this pressure range.

0.13 Supplemental Figure 13

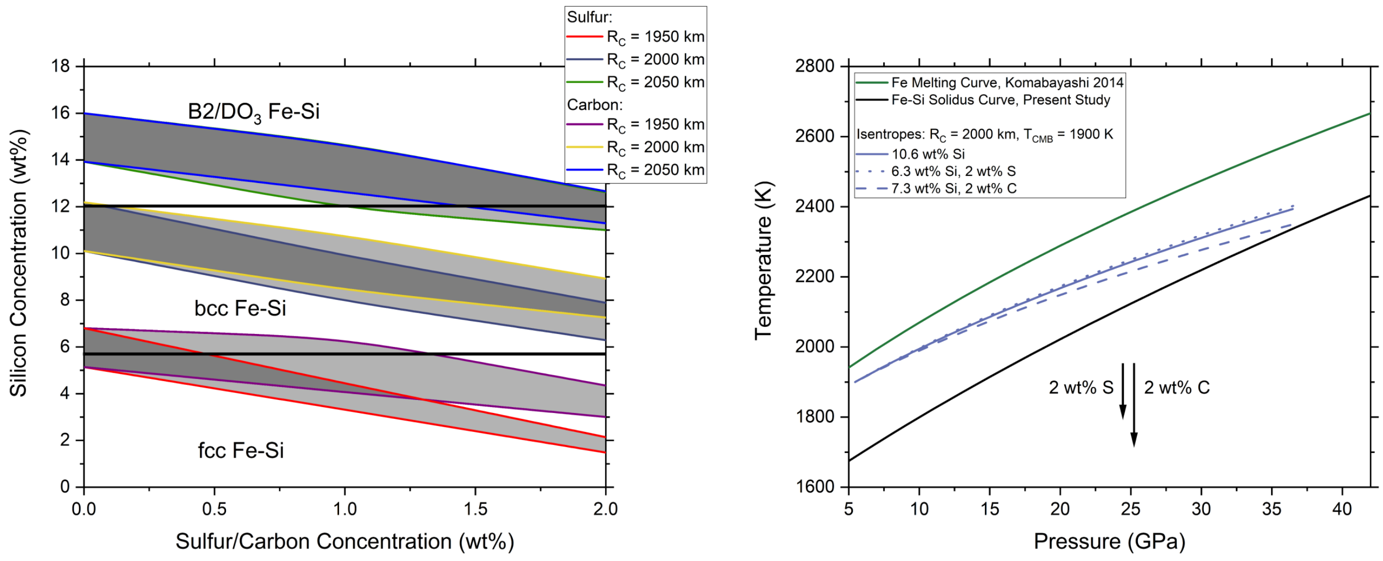

Figure 13: Left: Fe-Si-S and Fe-Si-C core compositions which are above solidus temperatures at P $< \sim$ 15 GPa, and below liquidus temperatures at P $< \sim$ 40 GPa relative to calculated isentropes. This range of conditions provides an estimate of bulk core compositions compatible with an active dynamo driven by compositional convection. Right: Variation of isentropic temperature profile with C or S at constant T_{CMB} and R_C. Arrows indicate the estimated effect of S/C on the solidus temperatures of Fe after[22,23,24].

Bibliography

[1] Jung-Fu Lin, Dion L. Heinz, Andrew J. Campbell, James M. Devine, and Guoyin Shen. Iron-silicon alloy in Earth's core? *Science*, 295(5553):313–315, 2002. doi: 10.1126/science.1066932.

[2] Rebecca A. Fischer, Andrew J. Campbell, Daniel M. Reaman, Noah A. Miller, Dion L. Heinz, Przymyslaw Dera, and Vitali B. Prakapenka. Phase relations in the Fe-FeSi system at high pressures and temperatures. *Earth and Planetary Science Letters*, 373:54 – 64, 2013. ISSN 0012-821X. doi: https://doi.org/10.1016/j.epsl.2013.04.035.

[3] Noriyoshi Tsujino, Yu Nishihara, Yoichi Nakajima, Eiichi Takahashi, Ken ichi Funakoshi, and Yuji Higo. Equation of state of γ-Fe: Reference density for planetary cores. *Earth and Planetary Science Letters*, 375: 244–253, 2013. doi: 10.1016/j.epsl.2013.05.040.

[4] Rachel A. Morrison, Jennifer M. Jackson, Wolfgang Sturhahn, Dongzhou Zhang, and Eran Greenberg. Equations of state and anisotropy of Fe-Ni-Si alloys. *Journal of Geophysical Research: Solid Earth*, in press:–, 04 2018.

[5] Rachel A. Morrison, Jennifer M. Jackson, Wolfgang Sturhahn, Jiyong Zhao, and Thomas S. Toellner. High pressure thermoelasticity and sound velocities of Fe-Ni-Si alloys. *Physics of the Earth and Planetary Interiors*, 294:106268, 2019. ISSN 0031-9201. doi: https://doi.org/10.1016/j.pepi.2019.05.011.

[6] E. Edmund, D. Antonangeli, F. Decremps, F. Miozzi, G. Morard, E. Boulard, A. N. Clark, S. Ayrinhac, M. Gauthier, M. Morand, and M. Mezouar. Velocity-Density Systematics of Fe-5wt%Si: Constraints on Si Content in the Earth's Inner Core. *Journal of Geophysical Research: Solid Earth*, 124, 2019. doi: 10.1029/2018JB016904.

[7] Eric Edmund, Francesca Miozzi, Guillaume Morard, Eglantine Boulard, Alisha Clark, Frédéric Decremps, Gaston Garbarino, Volodymyr Svitlyk, Mohamed Mezouar, and Daniele Antonangeli. Axial Compressibility and Thermal Equation of State of Hcp Fe–5wtNi–5wt *Minerals*, 10(2), 2020. ISSN 2075-163X. doi: 10.3390/min10020098.

[8] Rebecca A. Fischer, Andrew J. Campbell, Razvan Caracas, Daniel M. Reaman, Przymyslaw Dera, and Vitali B. Prakapenka. Equation of state and phase diagram of Fe-16Si alloy as a candidate component of Earth's core. *Earth and Planetary Science Letters*, 357-358:268 – 276, 2012. ISSN 0012-821X. doi: https://doi.org/10.1016/j.epsl.2012.09.022.

[9] Rebecca A. Fischer, Andrew J. Campbell, Razvan Caracas, Daniel M. Reaman, Dion L. Heinz, Przemyslaw Dera, and Vitali B. Prakapenka. Equations of state in the Fe-FeSi system at high pressures and temperatures. *Journal of Geophysical Research: Solid Earth*, 119(4):2810–2827, 2014. doi: 10.1002/2013JB010898.

[10] E. Edmund, D. Antonangeli, F. Decremps, G. Morard, S. Ayrinhac, M. Gauthier, E. Boulard, M. Mezouar, M. Hanfland, and N. Guignot. Structure and elasticity of cubic Fe-Si alloys at high pressures. *Phys. Rev. B*, 100:134105, Oct 2019. doi: 10.1103/PhysRevB.100.134105.

[11] Haruka Ozawa, Kei Hirose, Kyoko Yonemitsu, and Yasuo Ohishi. High-pressure melting experiments on Fe–Si alloys and implications for silicon as a light element in the core. *Earth and Planetary Science Letters*, 456: 47–54, 2016. doi: 10.1016/j.epsl.2016.08.042.

[12] Sarah M. Arveson, Jie Deng, Bijaya B. Karki, and Kanani K. M. Lee. Evidence for Fe-Si-O liquid immiscibility at deep Earth pressures. *Proceedings of the National Academy of Sciences*, 116(21):10238–10243, 2019. ISSN 0027-8424. doi: 10.1073/pnas.1821712116.

[13] Kei Hirose, Guillaume Morard, Ryosuke Sinmyo, Koichio Umemoto, John Hernlund, George Helffrich, and Stéphane Labrosse. Crystallization of silicon dioxide and compositional evolution of the Earth's core. *Nature*, 543:99–, February 2017.

[14] Dongyang Huang, James Badro, John Brodholt, and Yunguo Li. Ab Initio Molecular Dynamics Investigation of Molten Fe–Si–O in Earth's Core. *Geophysical Research Letters*, 46(12):6397–6405, 2019. doi: 10.1029/2019GL082722.

[15] Guillaume Morard, Denis Andrault, Nicolas Guignot, Julien Siebert, Gaston Garbarino, and Daniele Antonangeli. Melting of Fe–Ni–Si and Fe–Ni–S alloys at megabar pressures: implications for the core–mantle boundary temperature. *Physics and Chemistry of Minerals*, 38(10):767–776, Dec 2011. ISSN 1432-2021. doi: 10.1007/s00269-011-0449-9.

[16] Shigehiko Tateno, Yasuhiro Kuwayama, Kei Hirose, and Yasuo Ohishi. The structure of Fe–Si alloy in Earth's inner core. *Earth and Planetary Science Letters*, 418:11 – 19, 2015. ISSN 0012-821X. doi: https://doi.org/10.1016/j.epsl.2015.02.008.

[17] Hidetoshi Asanuma, Eiji Ohtani, Takeshi Sakai, Hidenori Terasaki, Seiji Kamada, Tadashi Kondo, and Takumi Kikegawa. Melting of iron-silicon alloy up to the core-mantle boundary pressure: implications to the thermal structure of the Earth's core. *Physics and Chemistry of Minerals*, 37(6):353–359, June 2010. ISSN 1432-2021.

[18] Yasuhiro Kuwayama and Kei Hirose. Phase relations in the system Fe-FeSi at 21 GPa. *American Mineralogist*, 89:273–276, 2004. doi: 10.2138/am-2004-2-303.

[19] Michael W. Guinan and Daniel N. Beshers. Pressure derivatives of the elastic constants of alpha-iron to 10 kbs. *Journal of Physics and Chemistry of Solids*, 29(3):541 – 549, 1968. ISSN 0022-3697. doi: https://doi.org/10.1016/0022-3697(68)90131-5.

[20] Shigehiko Tateno, Tetsuya Komabayashi, Kei Hirose, Naohisa Hirao, and Yasuo Ohishi. Static compression of B2 KCl to 230 GPa and its P-V-T equation of state. *American Mineralogist*, 104(5):718 – 723, 2019. doi: https://doi.org/10.2138/am-2019-6779.

[21] Anil K. Singh and Takemura Kenichi. Measurement and analysis of nonhydrostatic lattice strain component in niobium to 145 GPa under various fluid pressure-transmitting media. *Journal of Applied Physics*, 90(7): 3269–3275, 2001. doi: 10.1063/1.1397283.

[22] Bin Chen, Jie Li, and Steven A. Hauck II. Non-ideal liquidus curve in the Fe-S system and Mercury's snowing core. *Geophysical Research Letters*, 35(7), 2008. doi: https://doi.org/10.1029/2008GL033311.

[23] O.T. Lord, M.J. Walter, R. Dasgupta, D. Walker, and S.M. Clark. Melting in the Fe–C system to 70 GPa. *Earth and Planetary Science Letters*, 284(1):157–167, 2009. ISSN 0012-821X. doi: https://doi.org/10.1016/j.epsl.2009.04.017.

[24] G. Morard, D. Andrault, D. Antonangeli, Y. Nakajima, A.L. Auzende, E. Boulard, S. Cervera, A. Clark, O.T. Lord, J. Siebert, V. Svitlyk, G. Garbarino, and M. Mezouar. Fe–FeO and Fe–Fe3C melting relations at Earth's core–mantle boundary conditions: Implications for a volatile-rich or oxygen-rich core. *Earth and Planetary Science Letters*, 473:94 – 103, 2017. ISSN 0012-821X. doi: https://doi.org/10.1016/j.epsl.2017.05.024.
