# Supplementary material for: The Fe-FeSi phase diagram at Mercury’s core conditions
Source: Nat Commun. 2022 Jan 19;13:387. doi: 10.1038/s41467-022-27991-9 (PMC8770642; doi:10.1038/s41467-022-27991-9)
Supplement: Supplementary file 3 — Description of Additional Supplementary Information [file 41467_2022_27991_MOESM3_ESM.pdf]

Description of Additional Supplementary Information

Title: Supplementary Data 1

Description: Encloses the datasets used to produce the figures and results of the present work. The sheet entitled ‘Melting T and compositions’ shows the P-T conditions at and just below melting for the various alloys studied, in addition to the results of chemical analysis of high temperature run products. The sheet entitled ‘Observed solid phases’ contains the collected P-T points where the various Fe-Si alloy phase assemblages were observed. The sheet entitled ‘fcc and bcc volumes’ contains collected high P-T volumes of fcc and bcc Fe7Si.
